# Atmospheric Interactions and Cardiac Arrhythmias: Langrish et al. Respond

**DOI:** 10.1289/ehp.1409636R

**Published:** 2015-06-01

**Authors:** Jeremy P. Langrish, Jenny A. Bosson, Thomas Sandström, Anders Blomberg, David E. Newby, Nicholas L. Mills

**Affiliations:** 1 University/BHF Centre for Cardiovascular Science, University of Edinburgh, United Kingdom; 2 Department of Public Health and Clinical Medicine, Division of Medicine/Respiratory Medicine, Umeå University, Umeå, Sweden

We agree with Čulić’s argument (2015) that environmental influences on human health are complex and likely multifactorial. Exposure to indoor and ambient urban air pollution has been estimated to contribute to 7 million premature deaths each year, predominantly from cardiovascular and respiratory conditions (Lim et al. 2012). Associations between exposure and cardiovascular mortality and morbidity have been demonstrated for nitrogen dioxide, sulfur dioxide, ozone, carbon monoxide, and particulate matter (Brook et al. 2010), although the associations are strongest for fine and ultrafine particulate matter (Hoek et al. 2013).

Air pollution is extremely complex and consists not of single components in isolation but rather combinations of components. These constituent components interact with one another in the environment, which may alter potential toxicity and the subsequent health impacts. Meteorological factors such as wind speed and direction, humidity, atmospheric pressure, and temperature play an important part both in determining an individual’s exposure to ambient air pollution and in affecting the concentrations, chemical composition, and clearance of elements of the air pollution mixture. This is particularly true for the secondary pollutant ozone, which has a strong relationship with season and temperature (Langrish et al. 2010b). We agree that, in assessing the impact of ambient air pollution on public health, it is important to assess the air pollution mixture as a whole.

In our studies we used a controlled exposure facility to assess, in a robust and well-validated fashion (Langrish et al. 2010a), the contribution to potential arrhythmogenesis of individual air pollutants—diesel exhaust, wood smoke, ozone, and nitrogen dioxide—as well as ambient air pollution in Beijing, China (Langrish et al. 2014). Exposure to neither air pollutants in isolation nor ambient Beijing air pollution was associated with cardiac dysrhythmia in either patients with coronary heart disease or healthy volunteers. As such, our studies do not address the influence of meteorological conditions on an individual’s risk of cardiac arrhythmia; indeed, the meteorological conditions in Beijing were fairly constant throughout our studies (Langrish et al. 2009, 2012).

There is emerging evidence that cardiovascular morbidity and mortality is associated with meteorological and environmental conditions, and we agree with Čulić’s statement that further research on the health impacts of atmospheric factors is important both for public health and for better understanding the interaction between urban air pollution and external influences.
